# Supplementary material for: Molecular imaging with engineered physiology
Source: Nat Commun. 2016 Dec 2;7:13607. doi: 10.1038/ncomms13607 (PMC5146284; doi:10.1038/ncomms13607)
Supplement: Supplementary Information — Supplementary Figures 1-5 and Supplementary Tables 1-8. [file ncomms13607-s1.pdf]

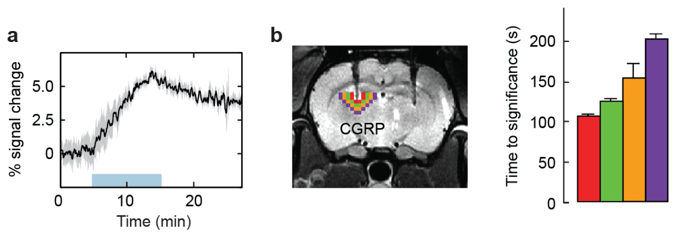

Supplementary Fig. 1. Time course of MRI signal during CGRP infusion in rat brain. (a) Time course of mean MRI signal change (black line) observed before, during (blue bar), and after 0.1 μL/min infusion of 100 nM wtCGRP into rat thalamus. Signals were averaged over six animals in 1.5 mm square regions of interest (ROIs) positioned around the infusion cannulae tips in each animal (cf. Fig. 2d); gray shading denotes standard errors as a function of time. **(b)** Time courses from multiple approximately concentric ROIs (diagrammed at left) were analyzed to determine whether hemodynamic MRI has sufficient spatial resolution to monitor convection of CGRP from the infusion site. For each ROI, the delay between the start of infusion and the observation of statistically significant signal changes from baseline was measured (color coded bars at right). The onset of significant signal changes increases monotonically with distance from the cannula tip, consistent with the expected spread of the imaging agent during infusion. Times to significance observed in peripheral ROIs (green, brown, and purple) were all significantly longer than the time to significance observed for the innermost ROI (red, all pairwise *t*-tests $p < 0.05$). Error bars denote SEM; all measurements $n = 6$.

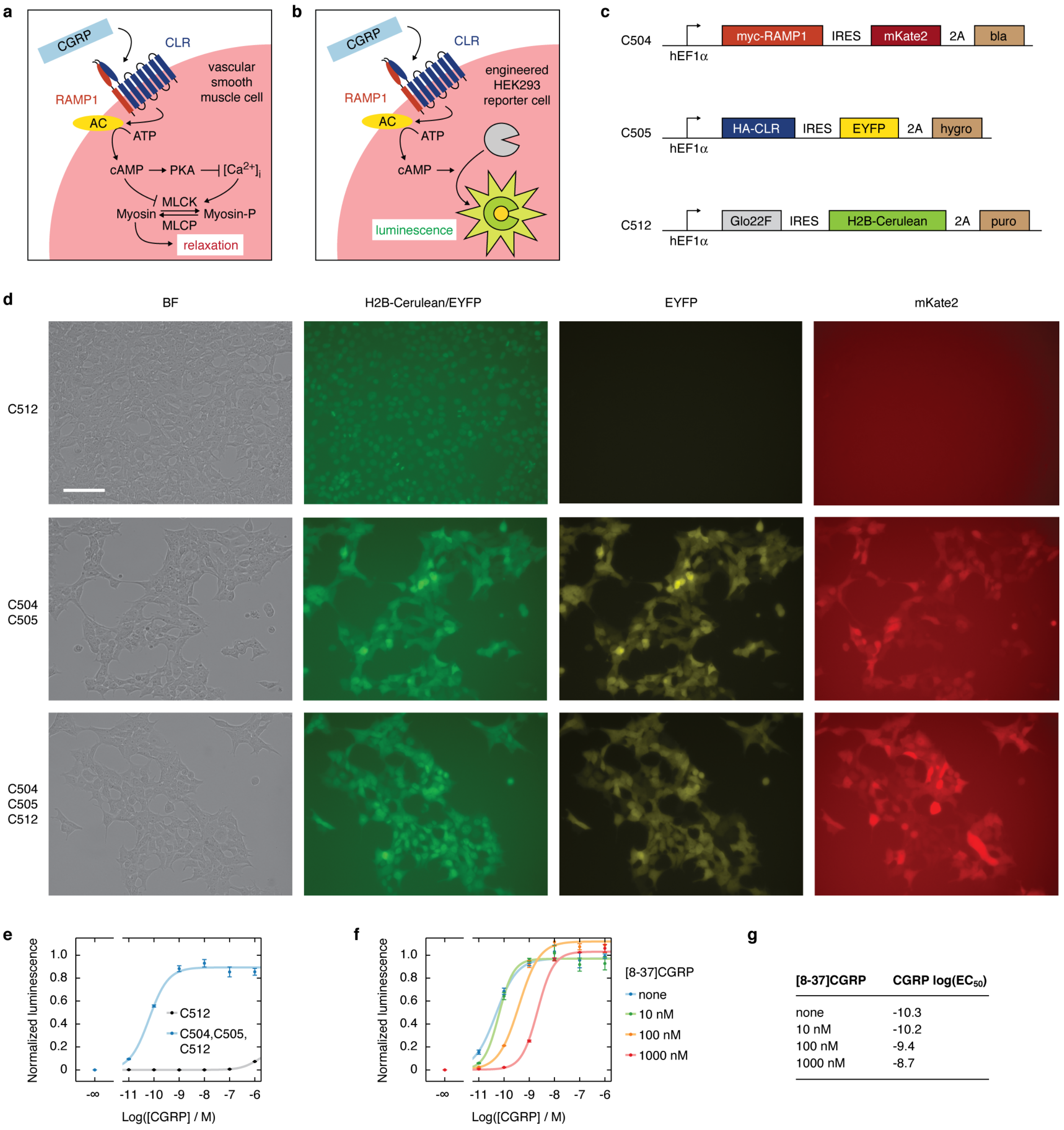

[8-37]CGRP	CGRP log(EC$_{50}$)
none	-10.3
10 nM	-10.2
100 nM	-9.4
1000 nM	-8.7

Supplementary Fig. 2. Cell-based bioassay for CGRP activity. (a) Mechanism of vasodilation by CGRP. CGRP peptide binds and activates the heterodimeric CGRP receptor (RAMP1:CLR) on vascular smooth muscle cells (VSMCs). Activation of adenylate cyclase (AC) results in cyclic adenosine monophosphate (cAMP) production that acts through protein kinase A (PKA)-dependent and independent pathways to inhibit myosin light chain kinase (MLCK), resulting in VSMC relaxtion and blood vessel dilation. **(b)** Engineered HEK293FT reporter cells provide a readout of cAMP accumulation upon activation of the CGRP receptor via a constitutively expressed, cAMP-responsive luciferase variant, Glo22F (Promega). **(c)** Lentiviral transgenes used to construct the CGRP reporter cell line. Full sequences provided in Supplementary Information.

(d) Fluorescence micrographs verifying expression of fluorescent reporters expressed by vectors encoding the Glo22F luciferase (C512) and the heterodimeric CGRP receptor components (C504 and C505) in cell lines transfected with constructs as noted at left. Color channels (labeled at top) depict bright field (BF) images, H2B-Cerulean (nuclear) with bleed-through from EYFP (cytosolic), EYFP alone, and mKate2. **(e-g)** Pharmacological validation of the reporter cell line, with error bars denoting SD of measurements performed in triplicate. **(e)** Dose-response curve for synthetic wild-type human alpha CGRP for reporter cell line infected with lentiviruses C504, C505, and C512 (blue) and for a cell line transfected with the cAMP reporter vector C512 but not the CGRP receptor vectors (gray). Luminescence values were normalized to the range evoked by buffer vs. 100 µM forskolin for each cell line. **(f)** Dose-reponse curves for CGRP in absence or presence of different concentrations of the competitive inhibitor [8-37]CGRP, a C-terminal fragment of CGRP. Luminescence values were normalized to the range obtained with buffer vs. 100 nM CGRP. **(g)** Tabulated potencies for CGRP in the presence of different [8-37]CGRP concentrations. SEM values were all less than 0.1 (n = 7).

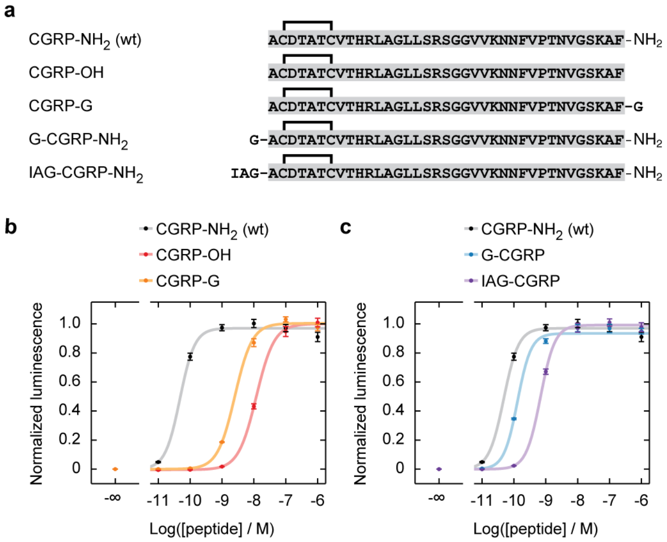

d

Peptide	Log(EC_{50})
CGRP-NH_2 (wt)	-10.3
CGRP-OH	-7.9
CGRP-G	-8.6
G-CGRP-NH_2	-9.9
IAG-CGRP-NH_2	-9.2

Supplementary Fig. 3. Impact of small terminal modifications on CGRP potency. (a) Sequence of peptide variants. -NH2 denotes an amidated C-terminus; CGRP-OH and CGRP-G contain a regular C-terminal carboxylate. Bioassays were performed with peptides serially diluted into water plus 0.1% CHAPS from 100 µM stock solutions in water or 50% DMSO. Error bars denote SD of measurements performed in triplicate. **(b)** Dose-response curves for C-terminally modified CGRP variants. Values normalized to the range obtained with buffer vs. 100 nM wtCGRP stimulation. **(c)** Dose-response curves for N-terminally modified CGRP variants. **(d)** Half maximal potencies, presented as log(EC_{50}), determined from the dose-response data in panels b and c. Standard errors were all less than 0.1 (n = 7).

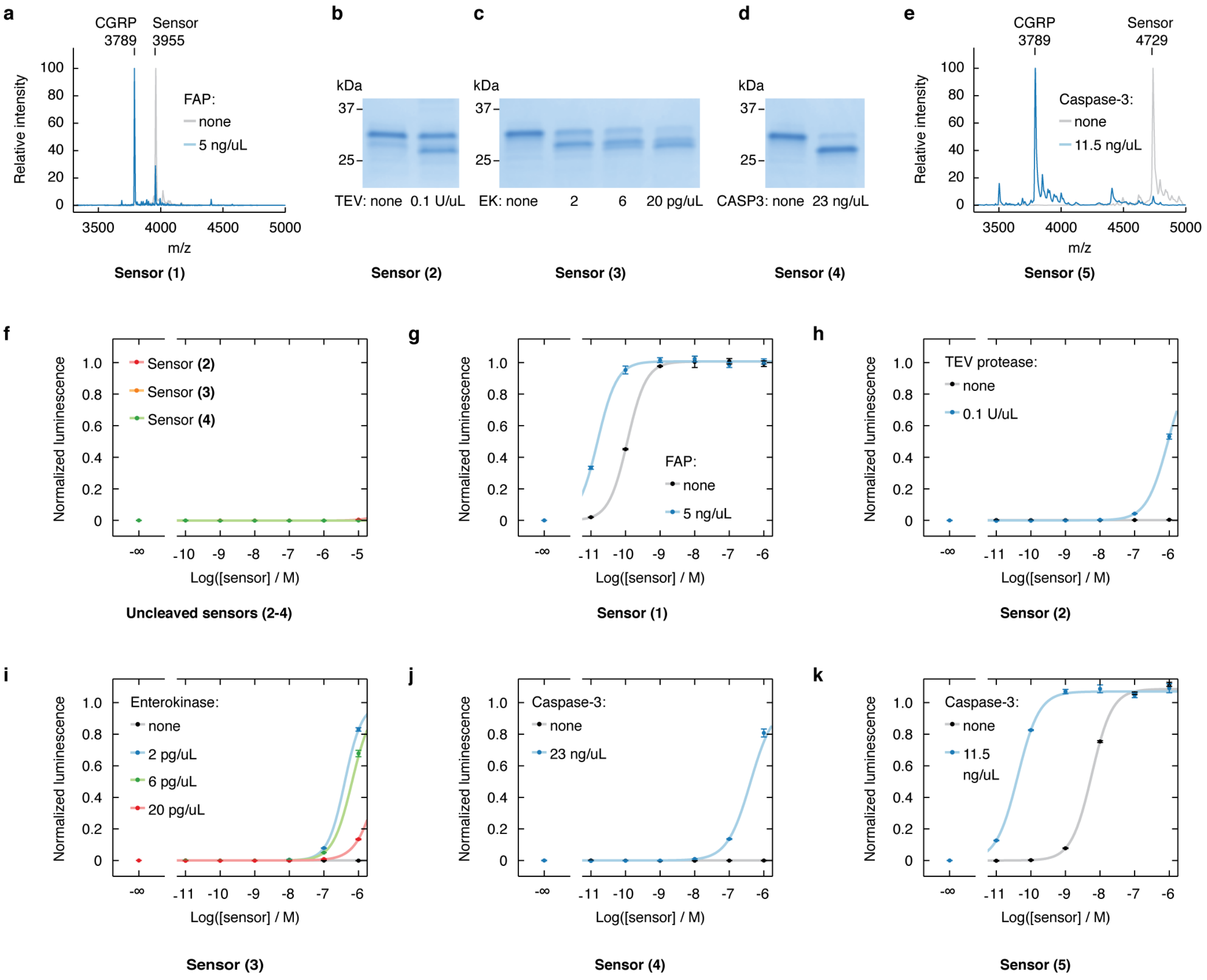

Supplementary Fig. 4. Proteolytic cleavage of candidate sensors and assessment by bioassay. (a-e) Cleavage reaction products after incubation of 10 µM sensor with or without enzyme for 2 h at 37 °C. **(a)** MALDI mass spectrum of sensor (1) after incubation with or without FAP shows near-complete cleavage and liberation of free CGRP. **(b)** SDS-PAGE analysis of sensor (2) after incubation with or without TEV protease shows substantial but not complete cleavage. **(c)** SDS-PAGE analysis of sensor (3) after incubation with different concentrations of EK light chain shows increasing cleavage with increasing concentrations. **(d)** SDS-PAGE analysis of sensor (4) after incubation with or without caspase-3 shows near-complete cleavage. **(e)** MALDI mass spectrum of sensor (5) after incubation with or without caspase-3 shows complete cleavage and liberation of free CGRP. **(f-k)** Dose-response curves of uncleaved and cleaved sensors in cell-based bioassay. All measurements were normalized to blank=0 and 100 µM wtCGRP = 1.0. **(f)** Dose-response curves for Sensors (2-4) at final assay concentrations up to 10 uM (addition of 10 µL of 100 µM solution to 90 µL medium per well in bioassay) show no receptor activation by uncleaved GFP-linker-CGRP-G sensors even at 10-fold higher concentrations than were used in cleavage assays (g-k). **(g-k)** Dose-response curves for reaction products from (a-e). Error bars in (f-k) denote SD of measurements performed in triplicate.

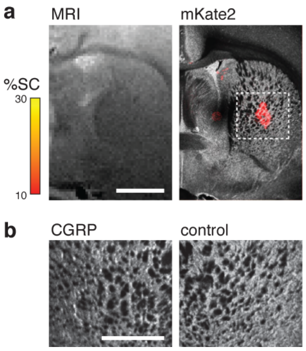

Supplementary Fig. 5. MRI and histological signals from injected control cells. (a) Comparison of MRI (left) and optical microscopy (right) images of a field of view near injection of control HEK293FT cells in an individual animal, paralleling the data in main text Fig. 4e. The MRI scan shows no colored voxels overlaid on the grayscale anatomical image because MRI signal changes over 10% were not observed within a day of control cell implantation; this is in contrast to MRI results from CGRP-expressing cells in Fig. 4e. The histological image shows a bright field grayscale image, with mKate fluorescence in red overlaid to indicate the distribution of injected cells. Scale bar = 2 mm. **(b)** Close-ups of bright field images in the neighborhood of cell implantation on CGRP-expressing (left) and control cell (right) injection sites in a representative animal, showing normal patch-matrix striatal structural organization on both sides. The field of view corresponds to the rectangular box in panel a (right). Scale bar = 1 mm.

Supplementary Table 1. Catalog of plasmids used in this study

ID	Name	Source
Z503	pEXPR-T7-cfSGFP2-DEVD-CGRP-G	This study
Z507	pEXPR-T7-cfSGFP2-DDDDK-CGRP-G	This study
Z508	pEXPR-T7-cfSGFP2-ENLYFQG-CGRP-G	This study
C501	pLV-hEF1a-mKate-2A-bla	This study
C503	pLV-hEF1a-prepro-CGRP-IRES-mKate-2A-bla	This study
C504	pLV-hEF1a-myc-RAMP1-IRES-mKate-2A-bla	This study
C505	pLV-hEF1a-HA-CALCRL-IRES-EYFP-2A-Hygro	This study
C512	pLV-hEF1a-Glo22F-IRES-H2B-Cerulean-2A-puro	This study
X106	pMD2.G	Addgene 12259
X107	psPAX2	Addgene 12260
X108	pLenti X1 Zeo DEST (668-1)	Addgene 17299
X109	pEF-ENTR A (696-6)	Addgene 17427

Supplementary Table 2. Full sequence for bacterial expression plasmid Z503 (pEXPR-T7-cfSGFP2-DEVD-CGRP-G)

color coding key:

cfSGFP2: cysteine-free GFP variant (with N-terminal StrepII tag underlined)
Cleavable linker
haCGRP-Gly: Human alpha CGRP extended with one C-terminal glycine residue
Kanamycin resistance gene
pBR322 origin of replication

sequence:

```
GCTCAGAGGATCGAGATCTCGATCCCGCGAAATTAATACGACTCACTATAGGGAGAGCCACAACGGTTTCCCTCTAGAAATAATTT
TGTTTAACTTTAAGAAGGAGATATACATATGAGCGCGTGGAGCCATCCGCAGTTTGAAAAAGGTGCTGTATCCAAGGGCGAAGAGC
TGTTCACGGGCGTAGTACCGATTTTGGTGGAGCTGGACGGCGATGTGAACGGCCATAAATTTAGTGTGAGCGGCGAAGGTGAGGGC
GATGCGACATATGGCAAACTGACCCTGAAATTTATTAGTACCACCGGTAAACTGCCAGTGCCTTGGCCGACGCTGGTTACCACCCT
GACCTACGGTGTGCAAATGTTCGCTCGCTACCCCGACCATATGAAACAGCATGACTTCTTCAAGAGCGCAATGCCGGAAGGTTACG
TGCAGGAGCGTACGATTTTTTTCAAAGATGATGGGAATTACAAGACACGTGCGGAAGTGAAATTCGAGGGCGATACGCTGGTTAAT
CGCATCGAACTGAAAGGCATCGACTTCAAAGAAGATGGTAACATTCTGGGTCACAAGCTTGAATACAACTACAACTCACATAACGT
ATATATCACAGCAGATAAGCAGAAGAATGGTATTAAGGCGAATTTCAAGATCCGTCACAATATTGAGGATGGCGGTGTTCAGTTAG
CTGATCATTATCAACAGAACACCCCTATTGGAGATGGCCCAGTGTTGCTGCCAGATAATCATTACCTGTCTACCCAGAGCAAACTG
AGCAAAGACCCTAATGAGAAACGTGATCATATGGTCTTACTGGAGTTCGTTACAGCGGCGGGCATTACGCTGGGTATGGATGAGCT
GTATAAGGGTGGCTCAGGTGATGAAGTAGATGCTTGTGACACAGCCACCTGTGTCACCCACCGCCTGGCCGGTCTGCTGTCTCGCT
CGGGTGGCGTTGTTAAAAACAATTTTGTGCCAACCAATGTTGGTTCAAAAGCGTTTGGCTAACAAAGCCCGAAAGGAAGCTGAGTT
GGCTGCTGCCACCGCTGAGCAATAACTAGCATAACCCCTTGGGGCCTCTAAACGGGTCTTGAGGGGTTTTTTGCTGAAAGGAGGAA
CTATATCCGGATATCCACAGGACGGAGGTAGCTCTGGCCCGTGTCTCAAAATCTCTGATGTTACATTGCACAAGATAAAAATATAT
CATCATGAACAATAAAACTGTCTGCTTACATAAACAGTAATACAAGGGGTGTTATGAGCCATATTCAACGGGAAACGTCGAGGCCG
CGATTAAATTCCAACATGGATGCTGATTTATATGGGTATAAATGGGCTCGCGATAATGTCGGGCAATCAGGTGCGACAATCTATCG
CTTGTATGGGAAGCCCGATGCGCCAGAGTTGTTTCTGAAACATGGCAAAGGTAGCGTTGCCAATGATGTTACAGATGAGATGGTCA
GACTAAACTGGCTGACGGAATTTATGCCTCTTCCGACCATCAAGCATTTTATCCGTACTCCTGGTGATGCATGGTTACTCACCACT
GCGATCCCCGGAAAAACAGCATTCCAGGTATTAGAAGAATATCCTGATTCAGGTGAAAATATTGTTGATGCGCTGGCAGTGTTCCT
GCGCCGGTTGCATTCGATTCCTGTTTGTAATTGTCCTTTTAACAGCGATCGCGTATTTCGCCTCGCTCAGGCGCAATCACGAATGA
ATAACGGTTTGGTTGATGCGAGTGATTTTGATGACGAGCGTAATGGCTGGCCTGTTGAACAAGTCTGGAAAGAAATGCATAAACTT
TTGCCATTCTCACCGGATTCAGTCGTCACTCATGGTGATTTCTCACTTGATAACCTTATTTTTGACGAGGGGAAATTAATAGGTTG
TATTGATGTTGGACGAGTCGGAATCGCAGACCGATACCAGGATCTTGCCATCCTATGGAACTGCCTCGGTGAGTTTTCTCCTTCAT
TACAGAAACGGCTTTTTCAAAAATATGGTATTGATAATCCTGATATGAATAAATTGCAGTTTCATTTGATGCTCGATGAGTTTTTC
TAATCAGAATTGGTTAATTGGTTGTAACATTATTCAGATTGGGCCCCGTTCCACTGAGCGTCAGACCCCGTAGAAAAGATCAAAGG
ATCTTCTTGAGATCCTTTTTTTCTGCGCGTAATCTGCTGCTTGCAAACAAAAAAACCACCGCTACCAGCGGTGGTTTGTTTGCCGG
ATCAAGAGCTACCAACTCTTTTTCCGAAGGTAACTGGCTTCAGCAGAGCGCAGATACCAAATACTGTTCTTCTAGTGTAGCCGTAG
TTAGGCCACCACTTCAAGAACTCTGTAGCACCGCCTACATACCTCGCTCTGCTAATCCTGTTACCAGTGGCTGCTGCCAGTGGCGA
TAAGTCGTGTCTTACCGGGTTGGACTCAAGACGATAGTTACCGGATAAGGCGCAGCGGTCGGGCTGAACGGGGGGTTCGTGCACAC
AGCCCAGCTTGGAGCGAACGACCTACACCGAACTGAGATACCTACAGCGTGAGCTATGAGAAAGCGCCACGCTTCCCGAAGGGAGA
AAGGCGGACAGGTATCCGGTAAGCGGCAGGGTCGGAACAGGAGAGCGCACGAGGGAGCTTCCAGGGGGAAACGCCTGGTATCTTTA
TAGTCCTGTCGGGTTTCGCCACCTCTGACTTGAGCGTCGATTTTTGTGATGCTCGTCAGGGGGGCGGAGCCTATGGAAAAACGCCA
GCAACGCGGCCTTTTTACGGTTCCTGGCCTTTTGCTGGCCTTTTGCTCACATGTTCTTTCCTGCGTTATCCCCTGATTCTGTGGAT
AACCGTATTACCGCTAGCATGGATCTCGGGGACGTCTAACTACTAAGCGAGAGTAGGGAACTGCCAGGCATCAAATAAAACGAAAG
GCCCAGTCTTCCGACTGAGCCTTTCGTTTTATCTGTTGTTTGTCGGTGAACGCTCTCCTGAGTAGGACAAATCCGCCGGGAGCGGA
TTTGAACGTTGTGAAGCAACGGCCCGGAGGGTGGCGGGCAGGACGCCCGCCATAAACTGCCAGGCATCAAACTAAGCAGAAGGCCA
TCCTGACGGATGGCCTTTTTGCGTTTCTACAAACTCTTCCTGTTAGTTAGTTACTTAA
```

Supplementary Table 3. DNA and amino acid sequence of cleavable linkers for all bacterial expression plasmids

Z503 (pEXPR-T7-cfSGFP2-DEVD-CGRP-G)	GGTGGCTCAGGTGATGAAGTAGAT<br>G G S G D E V D
Z507 (pEXPR-T7-cfSGFP2-DDDDK-CGRP-G)	GGTGGCTCAGGTGACGATGATGACAAG<br>G G S G D D D D K
Z508 (pEXPR-T7-cfSGFP2-ENLYFQG-CGRP-G)	GGTGGCTCAGGTGAAAACTTGTATTTCCAGGGT<br>G G S G E N L Y F Q G

Supplementary Table 4. Full sequence of lentiviral plasmid C501 (pLV-hEF1a- mKate-2A-bla)

color coding key:

hEF1a: Human elongation factor 1 alpha promoter
Transgene cassette: mKate2: Red fluorescent protein
2A: Autoproteolytic viral amino acid sequence
Bla: Blasticidin resistance gene
delta_U3 / 3’LTR: HIV 3’LTR with deletion in the U3 region
AmpR: Ampicillin resistance gene
pUC origin of replication
RSV / 5’LTR: Rous Sarcoma Virus / HIV 5'LTR hybrid promoter
Psi: Lentiviral Psi packaging sequence
RRE: Rev response element
CPPT: Central polypurine tract

sequence:

CTTTGCAGCTAATGGACCTTCTAGGTCTTGAAAGGAGTGGGAATTGGCTCCGGTGCCCGTCAGTGGGCAGAGCGCACATCGCCCAC
AGTCCCCGAGAAGTTGGGGGGAGGGGTCGGCAATTGAACCGGTGCCTAGAGAAGGTGGCGCGGGGTAAACTGGGAAAGTGATGTCG
TGTACTGGCTCCGCCTTTTTCCCGAGGGTGGGGGAGAACCGTATATAAGTGCAGTAGTCGCCGTGAACGTTCTTTTTCGCAACGGG
TTTGCCGCCAGAACACAGGTAAGTGCCGTGTGTGGTTCCCGCGGGCCTGGCCTCTTTACGGGTTATGGCCCTTGCGTGCCTTGAAT
TACTTCCACCTGGCTGCAGTACGTGATTCTTGATCCCGAGCTTCGGGTTGGAAGTGGGTGGGAGAGTTCGAGGCCTTGCGCTTAAG
GAGCCCCTTCGCCTCGTGCTTGAGTTGAGGCCTGGCCTGGGCGCTGGGGCCGCCGCGTGCGAATCTGGTGGCACCTTCGCGCCTGT
CTCGCTGCTTTCGATAAGTCTCTAGCCATTTAAAATTTTTGATGACCTGCTGCGACGCTTTTTTTCTGGCAAGATAGTCTTGTAAA
TGCGGGCCAAGATCTGCACACTGGTATTTCGGTTTTTGGGGCCGCGGGCGGCGACGGGGCCCGTGCGTCCCAGCGCACATGTTCGG
CGAGGCGGGGCCTGCGAGCGCGGCCACCGAGAATCGGACGGGGGTAGTCTCAAGCTGGCCGGCCTGCTCTGGTGCCTGGCCTCGCG
CCGCCGTGTATCGCCCCGCCCTGGGCGGCAAGGCTGGCCCGGTCGGCACCAGTTGCGTGAGCGGAAAGATGGCCGCTTCCCGGCCC
TGCTGCAGGGAGCTCAAAATGGAGGACGCGGCGCTCGGGAGAGCGGGCGGGTGAGTCACCCACACAAAGGAAAAGGGCCTTTCCGT
CCTCAGCCGTCGCTTCATGTGACTCCACGGAGTACCGGGCGCCGTCCAGGCACCTCGATTAGTTCTCGAGCTTTTGGAGTACGTCG
TCTTTAGGTTGGGGGGAGGGGTTTTATGCGATGGAGTTTCCCCACACTGAGTGGGTGGAGACTGAAGTTAGGCCAGCTTGGCACTT
GATGTAATTCTCCTTGGAATTTGCCCTTTTTGAGTTTGGATCTTGGTTCATTCTCAAGCCTCAGACAGTGGTTCAAAGTTTTTTTC
TTCCATTTCAGGTGTCGTGAGGAATTAGCTTGGTACTAATACGACTCACTATAGCCTGGCCACCATGGTGAGCGAGCTGATTAAGG
AGAACATGCACATGAAGCTGTACATGGAGGGCACCGTGAACAACCACCACTTCAAGTGCACATCCGAGGGCGAAGGCAAGCCCTAC
GAGGGCACCCAGACCATGAGAATCAAGGCGGTCGAGGGCGGCCCTCTCCCCTTCGCCTTCGACATCCTGGCTACCAGCTTCATGTA
CGGCAGCAAAACCTTCATCAACCACACCCAGGGCATCCCCGACTTCTTTAAGCAGTCCTTCCCCGAGGGCTTCACATGGGAGAGAG
TCACCACATACGAAGATGGGGGCGTGCTGACCGCTACCCAGGACACCAGCCTCCAGGACGGCTGCCTCATCTACAACGTCAAGATC
AGAGGGGTGAACTTCCCATCCAACGGCCCTGTGATGCAGAAGAAAACACTCGGCTGGGAGGCCTCCACCGAGACACTGTACCCCGC
TGACGGCGGCCTGGAAGGCAGAGCCGACATGGCCCTGAAGCTCGTGGGCGGGGGCCACCTGATCTGCAACCTTAAGACCACATACA
GATCCAAGAAACCCGCTAAGAACCTCAAGATGCCCGGCGTCTACTATGTGGACAGGAGACTGGAAAGAATCAAGGAGGCCGACAAA
GAGACATACGTCGAGCAGCACGAGGTGGCTGTGGCCAGATACTGCGACCTCCCTAGCAAACTGGGGCACAAACTTAATTCCGCTGA
GGGCCGCGGCAGCCTGCTGACCTGCGGCGACGTGGAGGAAAACCCAGGCCCAATGGCTAAGCCTTTGTCTCAAGAAGAATCCACCC
TCATTGAAAGAGCAACGGCTACAATCAACAGCATCCCCATCTCTGAAGACTACAGCGTCGCCAGCGCAGCTCTCTCTAGCGACGGC
CGCATCTTCACTGGTGTCAATGTATATCATTTTACTGGGGGACCTTGTGCAGAACTCGTGGTGCTGGGCACTGCTGCTGCTGCGGC
AGCTGGCAACCTGACTTGTATCGTCGCGATCGGAAATGAGAACAGGGGCATCTTGAGCCCCTGCGGACGGTGCCGACAGGTGCTTC
TCGATCTGCATCCTGGGATCAAAGCCATAGTGAAGGACAGTGATGGACAGCCGACGGCAGTTGGGATTCGTGAATTGCTGCCCTCT
GGTTATGTGTGGGAGGGATAAGGGACAGGTGATATCCAGCACAGTGGCGGCCGCTCGACAATCAACCTCTGGATTACAAAATTTGT
GAAAGATTGACTGGTATTCTTAACTATGTTGCTCCTTTTACGCTATGTGGATACGCTGCTTTAATGCCTTTGTATCATGCTATTGC
TTCCCGTATGGCTTTCATTTTCTCCTCCTTGTATAAATCCTGGTTGCTGTCTCTTTATGAGGAGTTGTGGCCCGTTGTCAGGCAAC
GTGGCGTGGTGTGCACTGTGTTTGCTGACGCAACCCCCACTGGTTGGGGCATTGCCACCACCTGTCAGCTCCTTTCCGGGACTTTC
GCTTTCCCCCTCCCTATTGCCACGGCGGAACTCATCGCCGCCTGCCTTGCCCGCTGCTGGACAGGGGCTCGGCTGTTGGGCACTGA
CAATTCCGTGGTGTTGTCGGGGAAGCTGACGTCCTTTCCATGGCTGCTCGCCTGTGTTGCCACCTGGATTCTGCGCGGGACGTCCT
TCTGCTACGTCCCTTCGGCCCTCAATCCAGCGGACCTTCCTTCCCGCGGCCTGCTGCCGGCTCTGCGGCCTCTTCCGCGTCTTCGC
CTTCGCCCTCAGACGAGTCGGATCTCCCTTTGGGCCGCCTCCCCGCCTGGAATTCTGCAGATATCCGGTTAGTAATGAGTTTGGAA
TTAATTCTGTGGAATGTGTGTCAGTTAGGGTGTGGAAAGTCCCCAGGCTCCCCAGGCAGGCAGAAGTATGCAAAGCATGCATCTCA
ATTAGTCAGCAACCAGGTGTGGAAAGTCCCCAGGCTCCCCAGCAGGCAGAAGTATGCAAAGCATGCATCTCAATTAGTCAGCAACC
ATAGTCCCGCCCCTAACTCCGCCCATCCCGCCCCTAACTCCGCCCAGTTCCGCCCATTCTCCGCCCCATGGCTGACTAATTTTTT
TATTTATGCAGAGGCCGAGGCCGCCTCTGCCTCTGAGCTATTCCAGAAGTAGTGAGGAGGCTTTTTTGGAGGCCTAGGCTTTTGCA
AAAAGCTCCCCCTGTTGACAATTAATCATCGGCATAGTATATCGGCATAGTATAATACGACAAGGTGAGGAACTAAACCATGGCCA

AGTTGACCAGTGCCGTTCCGGTGCTCACCGCGCGCGACGTCGCCGGAGCGGTCGAGTTCTGGACCGACCGGCTCGGGTTCTCCCGG
GACTTCGTGGAGGACGACTTCGCCGGTGTGGTCCGGGACGACGTGACCCTGTTCATCAGCGCGGTCCAGGACCAGGTGGTGCCGGA
CAACACCCTGGCCTGGGTGTGGGTGCGCGGCCTGGACGAGCTGTACGCCGAGTGGTCGGAGGTCGTGTCCACGAACTTCCGGGACG
CCTCCGGGCCGGCCATGACCGAGATCGGCGAGCAGCCGTGGGGGCGGGAGTTCGCCCTGCGCGACCCGGCCGGCAACTGCGTGCAC
TTCGTGGCCGAGGAGCAGGACTGACACGTGCTACGAGATTTAAATGGTACCTTTAAGACCAATGACTTACAAGGCAGCTGTAGATC
TTAGCCACTTTTTAAAAGAAAAGGGGGGACTGGAAGGGCTAGCTCACTCCCAACGAAGACAAGATCTGCTTTTTGCTTGTACTGGG
TCTCTCTGGTTAGACCAGATCTGAGCCTGGGAGCTCTCTGGCTGCCTAGGGAACCCACTGCTTAAGCCTCAATAAAGCTTGCCTTG
AGTGCTTCAAGTAGTGTGTGCCCGTCTGTTGTGTGACTCTGGTAACTAGAGATCCCTCAGACCCTTTTAGTCAGTGTGGAAAATCT
CTAGCAGTAGTAGTTCATGTCATCTTATTATTCAGTATTTATAACTTGCAAAGAAATGAATATCAGAGAGTGAGAGGAACTTGTTT
ATTGCAGCTTATAATGGTTACAAATAAAGCAATAGCATCACAAATTTCACAAATAAAGCATTTTTTTCACTGCATTCTAGTTGTGG
TTTGTCCAAACTCATCAATGTATCTTATCATGTCTGGCTCTAGCTATCCCGCCCCTAACTCCGCCCAGTTCCGCCCATTCTCCGCC
CCATGGCTGACTAATTTTTTTTATTTATGCAGAGGCCGAGGCCGACGTGTGTTTCTTAGACGTCAGGTGGCACTTTTCGGGGAAAT
GTGCGCGGAACCCCTATTTGTTTATTTTTCTAAATACATTCAAATATGTATCCGCTCATGAGACAATAACCCTGATAAATGCTTCA
ATAATATTGAAAAAGGAAGAGTATGAGTATTCAACATTTCCGTGTCGCCCTTATTCCCTTTTTTGCGGCATTTTGCCTTCCTGTTT
TTGCTCACCCAGAAACGCTGGTGAAAGTAAAAGATGCTGAAGATCAGTTGGGTGCACGAGTGGGTTACATCGAACTGGATCTCAAC
AGCGGTAAGATCCTTGAGAGTTTTCGCCCCGAAGAACGTTTTCCAATGATGAGCACTTTTAAAGTTCTGCTATGTGGCGCGGTATT
ATCCCGTATTGACGCCGGGCAAGAGCAACTCGGTCGCCGCATACACTATTCTCAGAATGACTTGGTTGAGTACTCACCAGTCACAG
AAAAGCATCTTACGGATGGCATGACAGTAAGAGAATTATGCAGTGCTGCCATAACCATGAGTGATAACACTGCGGCCAACTTACTT
CTGACAACGATCGGAGGACCGAAGGAGCTAACCGCTTTTTTGCACAACATGGGGGATCATGTAACTCGCCTTGATCGTTGGGAACC
GGAGCTGAATGAAGCCATACCAAACGACGAGCGTGACACCACGATGCCTGTAGCAATGGCAACAACGTTGCGCAAACTATTAACTG
GCGAACTACTTACTCTAGCTTCCCGGCAACAATTAATAGACTGGATGGAGGCGGATAAAGTTGCAGGACCACTTCTGCGCTCGGCC
CTTCCGGCTGGCTGGTTTATTGCTGATAAATCTGGAGCCGGTGAGCGTGGGTCTCGCGGTATCATTGCAGCACTGGGGCCAGATGG
TAAGCCCTCCCGTATCGTAGTTATCTACACGACGGGGAGTCAGGCAACTATGGATGAACGAAATAGACAGATCGCTGAGATAGGTG
CCTCACTGATTAAGCATTGGTAACTGTCAGACCAAGTTTACTCATATATACTTTAGATTGATTTAAAACTTCATTTTTAATTTAAA
AGGATCTAGGTGAAGATCCTTTTTGATAATCTCATGACCAAAATCCCTTAACGTGAGTTTTCGTTCCACTGAGCGTCAGACCCCGT
AGAAAAGATCAAAGGATCTTCTTGAGATCCTTTTTTTCTGCGCGTAATCTGCTGCTTGCAAACAAAAAAACCACCGCTACCAGCGG
TGGTTTGTTTGCCGGATCAAGAGCTACCAACTCTTTTTCCGAAGGTAACTGGCTTCAGCAGAGCGCAGATACCAAATACTGTCCTT
CTAGTGTAGCCGTAGTTAGGCCACCACTTCAAGAACTCTGTAGCACCGCCTACATACCTCGCTCTGCTAATCCTGTTACCAGTGGC
TGCTGCCAGTGGCGATAAGTCGTGTCTTACCGGGTTGGACTCAAGACGATAGTTACCGGATAAGGCGCAGCGGTCGGGCTGAACGG
GGGGTTCGTGCACACAGCCCAGCTTGGAGCGAACGACCTACACCGAACTGAGATACCTACAGCGTGAGCTATGAGAAAGCGCCACG
CTTCCCGAAGGGAGAAAGGCGGACAGGTATCCGGTAAGCGGCAGGGTCGGAACAGGAGAGCGCACGAGGGAGCTTCCAGGGGGAAA
CGCCTGGTATCTTTATAGTCCTGTCGGGTTTCGCCACCTCTGACTTGAGCGTCGATTTTTGTGATGCTCGTCAGGGGGGCGGAGCC
TATGGAAAAACGCCAGCAACGCGGCCTTTTTACGGTTCCTGGCCTTTTGCTGGCCTTTTGCTCACATGTTCTTTCCTGCGTTATCC
CCTGATTCTGTGGATAACCGTATTACCGCCTTTGAGTGAGCTGATACCGCTCGCCGCAGCCGAACGACCGAGCGCAGCGAGTCAGT
GAGCGAGGAAGCGGAAGAGCGCCCAATACGCAAACCGCCTCTCCCCGCGCGTTGGCCGATTCATTAATGCAGCTGGCACGACAGGT
TTCCCGACTGGAAAGCGGGCAGTGAGCGCAACGCAATTAATGTGAGTTAGCTCACTCATTAGGCACCCCAGGCTTTACACTTTATG
CTTCCGGCTCGTATGTTGTGTGGAATTGTGAGCGGATAACAATTTCACACAGGAAACAGCTATGACCATGATTACGCCAAGCGCGC
AATTAACCCTCACTAAAGGGAACAAAAGCTGGAGCTGCAAGCTTAATGTAGTCTTATGCAATACTCTTGTAGTCTTGCAACATGGT
AACGATGAGTTAGCAACATGCCTTACAAGGAGAGAAAAAGCACCGTGCATGCCGATTGGTGGAAGTAAGGTGGTACGATCGTGCCT
TATTAGGAAGGCAACAGACGGGTCTGACATGGATTGGACGAACCACTGAATTGCCGCATTGCAGAGATATTGTATTTAAGTGCCTA
GCTCGATACAATAAACGGGTCTCTCTGGTTAGACCAGATCTGAGCCTGGGAGCTCTCTGGCTAACTAGGGAACCCACTGCTTAAGC
CTCAATAAAGCTTGCCTTGAGTGCTTCAAGTAGTGTGTGCCCGTCTGTTGTGTGACTCTGGTAACTAGAGATCCCTCAGACCCTTT
TAGTCAGTGTGGAAAATCTCTAGCAGTGGCGCCCGAACAGGGACCTGAAAGCGAAAGGGAAACCAGAGCTCTCTCGACGCAGGACT
CGGCTTGCTGAAGCGCGCACGGCAAGAGGCGAGGGGCGGCGACTGGTGAGTACGCCAAAAATTTTGACTAGCGGAGGCTAGAAGGA
GAGAGATGGGTGCGAGAGCGTCAGTATTAAGCGGGGGAGAATTAGATCGCGATGGGAAAAAATTCGGTTAAGGCCAGGGGGAAAGA
AAAAATATAAATTAAAACATATAGTATGGGCAAGCAGGGAGCTAGAACGATTCGCAGTTAATCCTGGCCTGTTAGAAACATCAGAA
GGCTGTAGACAAATACTGGGACAGCTACAACCATCCCTTCAGACAGGATCAGAAGAACTTAGATCATTATATAATACAGTAGCAAC
CCTCTATTGTGTGCATCAAAGGATAGAGATAAAAGACACCAAGGAAGCTTTAGACAAGATAGAGGAAGAGCAAAACAAAAGTAAGA
CCACCGCACAGCAAGCGGCCGCTGATCTTCAGACCTGGAGGAGGAGATATGAGGGACAATTGGAGAAGTGAATTATATAAATATAA
AGTAGTAAAAATTGAACCATTAGGAGTAGCACCCACCAAGGCAAAGAGAAGAGTGGTGCAGAGAGAAAAAAGAGCAGTGGGAATAG
GAGCTTTGTTCCTTGGGTTCTTGGGAGCAGCAGGAAGCACTATGGGCGCAGCCTCAATGACGCTGACGGTACAGGCCAGACAATTA
TTGTCTGGTATAGTGCAGCAGCAGAACAATTTGCTGAGGGCTATTGAGGCGCAACAGCATCTGTTGCAACTCACAGTCTGGGGCAT
CAAGCAGCTCCAGGCAAGAATCCTGGCTGTGGAAAGATACCTAAAGGATCAACAGCTCCTGGGGATTTGGGGTTGCTCTGGAAAAC
TCATTTGCACCACTGCTGTGCCTTGGAATGCTAGTTGGAGTAATAAATCTCTGGAACAGATTGGAATCACACGACCTGGATGGAGT
GGGACAGAGAAATTAACAATTACACAAGCTTAATACACTCCTTAATTGAAGAATCGCAAAACCAGCAAGAAAAGAATGAACAAGAA
TTATTGGAATTAGATAAATGGGCAAGTTTGTGGAATTGGTTTAACATAACAAATTGGCTGTGGTATATAAAATTATTCATAATGAT
AGTAGGAGGCTTGGTAGGTTTAAGAATAGTTTTTGCTGTACTTTCTATAGTGAATAGAGTTAGGCAGGGATATTCACCATTATCGT
TTCAGACCCACCTCCCAACCCCGAGGGGACCCGACAGGCCCGAAGGAATAGAAGAAGAAGGTGGAGAGAGAGACAGAGACAGATCC
ATTCGATTAGTGAACGGATCTCGACGGTTAACTTTTAAAAGAAAAGGGGGGATTGGGGGGTACAGTGCAGGGGAAAGAATAGTAGA
CATAATAGCAACAGACATACAAACTAAAGAATTACAAAAACAAATTACAAAAATTCAAAATTTTATTCCAGTGTGGTGGAATTCTG
CAGTCTGC

Supplementary Table 5. Sequence of transgene cassette for C503 (pLV-hEF1a-prepro-CGRP-IRES-mKate2-2A-bla)

color coding key:

prepro-CGRP: human prepro-CGRP alpha
IRES: Internal ribosome entry site
mKate2: Red fluorescent protein
2A: Autoproteolytic viral amino acid sequence
Bla: Blasticidin resistance gene

sequence:

ATGGGCTTCCAAAAGTTCTCCCCCTTCCTGGCTCTCAGCATCTTGGTCCTGTTGCAGGCAGGCAGCCTCCATGCAGCACCATTCAG
GTCTGCCCTGGAGAGCAGCCCAGCAGACCCGGCCACGCTCAGTGAGGACGAAGCGCGCCTCCTGCTGGCTGCACTGGTGCAGGACT
ATGTGCAGATGAAGGCCAGTGAGCTGGAGCAGGAGCAAGAGAGAGAGGGCTCCAGAATCATTGCCCAGAAGAGAGCCTGTGACACT
GCCACCTGTGTGACTCATCGGCTGGCAGGCTTGCTGAGCAGATCAGGGGGTGTGGTGAAGAACAACTTTGTGCCCACCAATGTGGG
TTCCAAAGCCTTTGGCAGGCGCCGCAGGGACCTTCAAGCCTGAGACTGGGATCCGCCCCTCTCCCTCCCCCCCCCCTAACGTTACT
GGCCGAAGCCGCTTGGAATAAGGCCGGTGTGCGTTTGTCTATATGTTATTTTCCACCATATTGCCGTCTTTTGGCAATGTGAGGGC
CCGGAAACCTGGCCCTGTCTTCTTGACGAGCATTCCTAGGGGTCTTTCCCCTCTCGCCAAAGGAATGCAAGGTCTGTTGAATGTCG
TGAAGGAAGCAGTTCCTCTGGAAGCTTCTTGAAGACAAACAACGTCTGTAGCGACCCTTTGCAGGCAGCGGAACCCCCCACCTGGC
GACAGGTGCCTCTGCGGCCAAAAGCCACGTGTATAAGATACACCTGCAAAGGCGGCACAACCCCAGTGCCACGTTGTGAGTTGGAT
AGTTGTGGAAAGAGTCAAATGGCTCTCCTCAAGCGTATTCAACAAGGGGCTGAAGGATGCCCAGAAGGTACCCCATTGTATGGGAT
CTGATCTGGGGCCTCGGTACACATGCTTTACATGTGTTTAGTCGAGGTTAAAAAAACGTCTAGGCCCCCCGAACCACGGGGACGTG
GTTTTCCTTTGAAAAACACGATGATAATATGGCCACAGCCACCATGGTGAGCGAGCTGATTAAGGAGAACATGCACATGAAGCTGT
ACATGGAGGGCACCGTGAACAACCACCACTTCAAGTGCACATCCGAGGGCGAAGGCAAGCCCTACGAGGGCACCCAGACCATGAGA
ATCAAGGCGGTCGAGGGCGGCCCTCTCCCCTTCGCCTTCGACATCCTGGCTACCAGCTTCATGTACGGCAGCAAAACCTTCATCAA
CCACACCCAGGGCATCCCCGACTTCTTTAAGCAGTCCTTCCCCGAGGGCTTCACATGGGAGAGAGTCACCACATACGAAGATGGGG
GCGTGCTGACCGCTACCCAGGACACCAGCCTCCAGGACGGCTGCCTCATCTACAACGTCAAGATCAGAGGGGTGAACTTCCCATCC
AACGGCCCTGTGATGCAGAAGAAAACACTCGGCTGGGAGGCCTCCACCGAGACACTGTACCCCGCTGACGGCGGCCTGGAAGGCAG
AGCCGACATGGCCCTGAAGCTCGTGGGCGGGGGCCACCTGATCTGCAACCTTAAGACCACATACAGATCCAAGAAACCCGCTAAGA
ACCTCAAGATGCCCGGCGTCTACTATGTGGACAGGAGACTGGAAAGAATCAAGGAGGCCGACAAAGAGACATACGTCGAGCAGCAC
GAGGTGGCTGTGGCCAGATACTGCGACCTCCCTAGCAAACTGGGGCACAAACTTAATTCCGCTGAGGGCCGCGGCAGCCTGCTGAC
CTGCGGCGACGTGGAGGAAAACCCAGGCCCAATGGCTAAGCCTTTGTCTCAAGAAGAATCCACCCTCATTGAAAGAGCAACGGCTA
CAATCAACAGCATCCCCATCTCTGAAGACTACAGCGTCGCCAGCGCAGCTCTCTCTAGCGACGGCCGCATCTTCACTGGTGTCAAT
GTATATCATTTTACTGGGGGACCTTGTGCAGAACTCGTGGTGCTGGGCACTGCTGCTGCTGCGGCAGCTGGCAACCTGACTTGTAT
CGTCGCGATCGGAAATGAGAACAGGGGCATCTTGAGCCCCTGCGGACGGTGCCGACAGGTGCTTCTCGATCTGCATCCTGGGATCA
AAGCCATAGTGAAGGACAGTGATGGACAGCCGACGGCAGTTGGGATTCGTGAATTGCTGCCCTCTGGTTATGTGTGGGAGGGATAA

Supplementary Table 6. Sequence of transgene cassette for C504 (pLV-hEF1a-myc-RAMP1-IRES-mKate-2A-bla)

color coding key:

myc-RAMP: Human receptor-activity modifying protein 1 preceded by the H. influenza hemagglutinin signal peptide and the c-myc epitope
IRES: Internal ribosome entry site
mKate2: Red fluorescent protein
2A: Autoproteolytic viral amino acid sequence
Bla: Blasticidin resistance gene

sequence:

ATGAAGACCATCCTGGCCCTGAGCACCTACATCTTCTGCCTGGTGTTCGCCGAGCAGAAGCTGATCAGCGAGGAGGACCTGGCCTG
CCAGGAGGCTAACTACGGTGCCCTCCTCCGGGAGCTCTGCCTCACCCAGTTCCAGGTAGACATGGAGGCCGTCGGGGAGACTCTGT
GGTGTGACTGGGGCAGGACCATCAGGAGCTACAGGGAGCTGGCCGACTGCACCTGGCACATGGCGGAGAAGCTGGGCTGCTTCTGG
CCCAATGCAGAGGTGGACAGGTTCTTCCTGGCAGTGCATGGCCGCTACTTCAGGAGCTGCCCCATCTCAGGCAGGGCCGTGCGGGA
CCCGCCCGGCAGCATCCTCTACCCCTTCATCGTGGTCCCCATCACGGTGACCCTGCTGGTGACGGCACTGGTGGTCTGGCAGAGCA
AGCGCACTGAGGGCATTGTGTAGGACTGGGATCCGCCCCTCTCCCTCCCCCCCCCCTAACGTTACTGGCCGAAGCCGCTTGGAATA
AGGCCGGTGTGCGTTTGTCTATATGTTATTTTCCACCATATTGCCGTCTTTTGGCAATGTGAGGGCCCGGAAACCTGGCCCTGTCT
TCTTGACGAGCATTCCTAGGGGTCTTTCCCCTCTCGCCAAAGGAATGCAAGGTCTGTTGAATGTCGTGAAGGAAGCAGTTCCTCTG
GAAGCTTCTTGAAGACAAACAACGTCTGTAGCGACCCTTTGCAGGCAGCGGAACCCCCCACCTGGCGACAGGTGCCTCTGCGGCCA
AAAGCCACGTGTATAAGATACACCTGCAAAGGCGGCACAACCCCAGTGCCACGTTGTGAGTTGGATAGTTGTGGAAAGAGTCAAAT
GGCTCTCCTCAAGCGTATTCAACAAGGGGCTGAAGGATGCCCAGAAGGTACCCCATTGTATGGGATCTGATCTGGGGCCTCGGTAC
ACATGCTTTACATGTGTTTAGTCGAGGTTAAAAAAACGTCTAGGCCCCCCGAACCACGGGGACGTGGTTTTCCTTTGAAAAACACG
ATGATAATATGGCCACAGCCACCATGGTGAGCGAGCTGATTAAGGAGAACATGCACATGAAGCTGTACATGGAGGGCACCGTGAAC
AACCACCACTTCAAGTGCACATCCGAGGGCGAAGGCAAGCCCTACGAGGGCACCCAGACCATGAGAATCAAGGCGGTCGAGGGCGG
CCCTCTCCCCTTCGCCTTCGACATCCTGGCTACCAGCTTCATGTACGGCAGCAAAACCTTCATCAACCACACCCAGGGCATCCCCG
ACTTCTTTAAGCAGTCCTTCCCCGAGGGCTTCACATGGGAGAGAGTCACCACATACGAAGATGGGGGCGTGCTGACCGCTACCCAG
GACACCAGCCTCCAGGACGGCTGCCTCATCTACAACGTCAAGATCAGAGGGGTGAACTTCCCATCCAACGGCCCTGTGATGCAGAA
GAAAACACTCGGCTGGGAGGCCTCCACCGAGACACTGTACCCCGCTGACGGCGGCCTGGAAGGCAGAGCCGACATGGCCCTGAAGC
TCGTGGGCGGGGGCCACCTGATCTGCAACCTTAAGACCACATACAGATCCAAGAAACCCGCTAAGAACCTCAAGATGCCCGGCGTC
TACTATGTGGACAGGAGACTGGAAAGAATCAAGGAGGCCGACAAAGAGACATACGTCGAGCAGCACGAGGTGGCTGTGGCCAGATA
CTGCGACCTCCCTAGCAAACTGGGGCACAAACTTAATTCCGCTGAGGGCCGCGGCAGCCTGCTGACCTGCGGCGACGTGGAGGAAA
ACCCAGGCCCAATGGCTAAGCCTTTGTCTCAAGAAGAATCCACCCTCATTGAAAGAGCAACGGCTACAATCAACAGCATCCCCATC
TCTGAAGACTACAGCGTCGCCAGCGCAGCTCTCTCTAGCGACGGCCGCATCTTCACTGGTGTCAATGTATATCATTTTACTGGGGG
ACCTTGTGCAGAACTCGTGGTGCTGGGCACTGCTGCTGCTGCGGCAGCTGGCAACCTGACTTGTATCGTCGCGATCGGAAATGAGA
ACAGGGGCATCTTGAGCCCCTGCGGACGGTGCCGACAGGTGCTTCTCGATCTGCATCCTGGGATCAAAGCCATAGTGAAGGACAGT
GATGGACAGCCGACGGCAGTTGGGATTCGTGAATTGCTGCCCTCTGGTTATGTGTGGGAGGGATAA

Supplementary Table 7. Sequence of transgene cassette for C505 (pLV-hEF1a-HA-CALCRL-IRES-EYFP-2A-Hygro)

color coding key:

HA-CALCRL: Human calcitonin receptor-like receptor preceded by the H. influenza hemagglutinin signal peptide and the influenza hemagglutinin antigen (HA) sequence
IRES: Internal ribosome entry site
EYFP: Enhanced yellow fluorescent protein
2A: Autoproteolytic viral amino acid sequence
Hygro: Hygromycin resistance gene

sequence:

```
ATGAAGACCATCCTGGCCCTGAGCACCTACATCTTCTGCCTGGTGTTCGCCTACCCCTACGACGTGCCCGTGTACGCCGCAGAATT
AGAAGAGAGTCCTGAGGACTCAATTCAGTTGGGAGTTACTAGAAATAAAATCATGACAGCTCAATATGAATGTTACCAAAAGATTA
TGCAAGACCCCATTCAACAAGCAGAAGGCGTTTACTGCAACAGAACCTGGGATGGATGGCTCTGCTGGAACGATGTTGCAGCAGGA
ACTGAATCAATGCAGCTCTGCCCTGATTACTTTCAGGACTTTGATCCATCAGAAAAAGTTACAAAGATCTGTGACCAAGATGGAAA
CTGGTTTAGACATCCAGCAAGCAACAGAACATGGACAAATTATACCCAGTGTAATGTTAACACCCACGAGAAAGTGAAGACTGCAC
TAAATTTGTTTTACCTGACCATAATTGGACACGGATTGTCTATTGCATCACTGCTTATCTCGCTTGGCATATTCTTTTATTTCAAG
AGCCTAAGTTGCCAAAGGATTACCTTACACAAAAATCTGTTCTTCTCATTTGTTTGTAACTCTGTTGTAACAATCATTCACCTCAC
TGCAGTGGCCAACAACCAGGCCTTAGTAGCCACAAATCCTGTTAGTTGCAAAGTGTCCCAGTTCATTCATCTTTACCTGATGGGCT
GTAATTACTTTTGGATGCTCTGTGAAGGCATTTACCTACACACACTCATTGTGGTGGCCGTGTTTGCAGAGAAGCAACATTTAATG
TGGTATTATTTTCTTGGCTGGGGATTTCCACTGATTCCTGCTTGTATACATGCCATTGCTAGAAGCTTATATTACAATGACAATTG
CTGGATCAGTTCTGATACCCATCTCCTCTACATTATCCATGGCCCAATTTGTGCTGCTTTACTGGTGAATCTTTTTTTCTTGTTAA
ATATTGTACGCGTTCTCATCACCAAGTTAAAAGTTACACACCAAGCGGAATCCAATCTGTACATGAAAGCTGTGAGAGCTACTCTT
ATCTTGGTGCCATTGCTTGGCATTGAATTTGTGCTGATTCCATGGCGACCTGAAGGAAAGATTGCAGAGGAGGTATATGACTACAT
CATGCACATCCTTATGCACTTCCAGGGTCTTTTGGTGTCTACCATTTTCTGCTTCTTTAATGGAGAGGTTCAAGCAATTCTGAGAA
GAAACTGGAATCAATACAAAATCCAATTTGGAAACAGCTTTTCCAACTCAGAAGCTCTTCGTAGTGCGTCTTACACAGTGTCAACA
ATCAGTGATGGTCCAGGTTATAGTCATGACTGTCCTAGTGAACACTTAAATGGAAAAAGCATCCATGATATTGAAAATGTTCTCTT
AAAACCAGAAAATTTATATAATTGAGACTGGGATCCGCCCCTCTCCCTCCCCCCCCCCTAACGTTACTGGCCGAAGCCGCTTGGAA
TAAGGCCGGTGTGCGTTTGTCTATATGTTATTTTCCACCATATTGCCGTCTTTTGGCAATGTGAGGGCCCGGAAACCTGGCCCTGT
CTTCTTGACGAGCATTCCTAGGGGTCTTTCCCCTCTCGCCAAAGGAATGCAAGGTCTGTTGAATGTCGTGAAGGAAGCAGTTCCTC
TGGAAGCTTCTTGAAGACAAACAACGTCTGTAGCGACCCTTTGCAGGCAGCGGAACCCCCCACCTGGCGACAGGTGCCTCTGCGGC
CAAAAGCCACGTGTATAAGATACACCTGCAAAGGCGGCACAACCCCAGTGCCACGTTGTGAGTTGGATAGTTGTGGAAAGAGTCAA
ATGGCTCTCCTCAAGCGTATTCAACAAGGGGCTGAAGGATGCCCAGAAGGTACCCCATTGTATGGGATCTGATCTGGGGCCTCGGT
ACACATGCTTTACATGTGTTTAGTCGAGGTTAAAAAAACGTCTAGGCCCCCCGAACCACGGGGACGTGGTTTTCCTTTGAAAAACA
CGATGATAATATGGCCACAGCCACCATGGTGAGCAAGGGCGAGGAGCTGTTCACCGGGGTGGTGCCCATCCTGGTCGAGCTGGACG
GCGACGTAAACGGCCACAAGTTCAGCGTGTCCGGCGAGGGCGAGGGCGATGCCACCTACGGCAAGCTGACCCTGAAGTTCATCTGC
ACCACCGGCAAGCTGCCCGTGCCCTGGCCCACCCTCGTGACCACCTTCGGCTACGGCCTGCAGTGCTTCGCCCGCTACCCCGACCA
CATGAAGCAGCACGACTTCTTCAAGTCCGCCATGCCCGAAGGCTACGTCCAGGAGCGCACCATCTTCTTCAAGGACGACGGCAACT
ACAAGACCCGCGCCGAGGTGAAGTTCGAGGGCGACACCCTGGTGAACCGCATCGAGCTGAAGGGCATCGACTTCAAGGAGGACGGC
AACATCCTGGGGCACAAGCTGGAGTACAACTACAACAGCCACAACGTCTATATCATGGCCGACAAGCAGAAGAACGGCATCAAGGT
GAACTTCAAGATCCGCCACAACATCGAGGACGGCAGCGTGCAGCTCGCCGACCACTACCAGCAGAACACCCCCATCGGCGACGGCC
CCGTGCTGCTGCCCGACAACCACTACCTGAGCTACCAGTCCAAGCTGAGCAAAGACCCCAACGAGAAGCGCGATCACATGGTCCTG
CTGGAGTTCGTGACCGCCGCCGGGATCACTCTCGGCATGGACGAGCTGTACAAGCAATTGGAGGGCCGCGGCAGCCTGCTGACCTG
CGGCGACGTGGAGGAAAACCCCGGCCCCATGAAAAAGCCTGAACTCACCGCGACGTCTGTCGAGAAGTTTCTGATCGAAAAGTTCG
ACAGCGTGTCCGACCTGATGCAGCTCTCGGAGGGCGAAGAATCTCGTGCTTTCAGCTTCGATGTAGGAGGGCGTGGATATGTCCTG
CGGGTAAATAGCTGCGCCGATGGTTTCTACAAAGATCGTTATGTTTATCGGCACTTTGCATCGGCCGCGCTCCCGATTCCGGAAGT
GCTTGACATTGGGGAATTCAGCGAGAGCCTGACCTATTGCATCTCCCGCCGTGCACAGGGTGTCACGTTGCAAGACCTGCCTGAAA
CCGAACTGCCCGCTGTTCTGCAGCCGGTCGCGGAGGCCATGGATGCGATCGCTGCGGCCGATCTTAGCCAGACGAGCGGGTTCGGC
CCATTCGGACCGCAAGGAATCGGTCAATACACTACATGGCGTGATTTCATATGCGCGATTGCTGATCCCCATGTGTATCACTCCTG
GCAAACTGTGATGGACGACACCGTCAGTGCGTCCGTCGCGCAGGCTCTCGATGAGCTGATGCTTTGGGCCGAGGACTGCCCCGAAG
TCCGGCACCTCGTGCACGCGGATTTCGGCTCCAACAATGTCCTGACGGACAATGGCCGCATAACAGCGGTCATTGACTGGAGCGAG
GCGATGTTCGGGGATTCCCAATACGAGGTCGCCAACATCTTCTTCTGGAGGCCGTGGTTGGCTTGTATGGAGCAGCAGACGCGCTA
CTTCGAGCGGAGGCATCCGGAGCTTGCAGGATCGCCGCGGCTCCGGGCGTATATGCTCCGCATTGGTCTTGACCAACTCTATCAGA
GCTTGGTTGACGGCAATTTCGATGATGCAGCTTGGGCGCAGGGTCGATGCGACGCAATCGTCCGATCCGGAGCCGGGACTGTCGGG
CGTACACAAATCGCCCGCAGAAGCGCGGCCGTCTGGACCGATGGCTGTGTAGAAGTACTCGCCGATAGTGGAAACCGACGCCCCAG
CACTCGTCCGGATCGGGAGATGGGGGAGGCTAACTGA
```

Supplementary Table 8. Sequence of transgene cassette for C512: pLV-hEF1a-Glo22F-IRES-H2B-Cerulean-2A-puro

color coding key:

Glo22F: Engineered cAMP-responsive luciferase (Promega Corp.)
IRES: Internal ribosome entry site
H2B-Cerulean: DNA binding domain of histone 2B fused to Cerulean
2A: Autoproteolytic viral amino acid sequence
Puro: Puromycin resistance gene

sequence:

ATGCCTGGCGCAGTAGGCAAGGTGGTGCCCTTCATCGAGGCTAAGGTGGTGGACTTGGACACTGGTAAGACACTGGGTGTGAACCA
GCGCGGCGAGCTGTGCGTCCGTGGCCCCATGATCATGAGCGGCTACGTTAACAACCCCGAGGCTACAAACGCTCTCATCGACAAGG
ACGGCTGGCTGCACAGCGGCGACATCGCCTACTGGGACGAGGACGAGCACTTCTTCATCGTGGACCGGCTGAAGAGCCTGATCAAA
TACAAGGGCTACCAGGTAGCCCCAGCCGAACTGGAGAGCATCCTGCTGCAACACCCCAACATCTTCGACGCCGGGGTCGCCGGCCT
GCCCGACGACGATGCCGGCGAGCTGCCCGCCGCAGTCGTCGTGCTGGAACACGGTAAAACCATGACCGAGAAGGAGATCGTGGACT
ATGTGGCCAGCCAGGTTACAACCGCCAAGAAGCTGCGCGGTGGTGTTGTGTTAGTGGACGAGGTGCCTAAAGGACTGACCGGCAAG
TTGGACGCCCGCAAGATCCGCGAGATTCTCATTAAGGCCAAGAAGGGATCCAATTGGGATTCTGGGTGCTCCAGAGAAGGTATGTA
TGAAAGCTTTATTGAGTCACTGCCATTCCTTAAATCTTTGGAGTTTTCTGCACGCCTGAAAGTAGTAGATGTGATAGGCACCAAAG
TATACAACGATGGAGAACAAATCATTGCTCAGGGAGATTCGGCTGATTCTTTTTTCATTATTGAATCTGGAGAAGTGAAAATTACT
ATGAAAAGAAAGGGTAAATCAGAAGTGGAAGAGAATGGTGCAGTAGAAATCGCTCGATGCTCGCGGGGACAGTACTTTGGAGAGCT
TGCCCTGGTAACTAACAAACCTCGAGCAGCTTCTGCCCACGCCATTGGGACTGTCAAATGTTTAGCAATGGATGTGCAAGCATTTG
AAAGGCTTCTGGGACCTTGCATGGAAATTATGAAAAGGAACATCGCTACCTATGAAGAACAGTTAGTTGCCCTGTATGGAACGAAC
ATGGATATTGTAGCCAAAAACATTAAGAAGGGCCCAGCGCCATTCTACCCACTCGAAGACGGGACCGCCGGCGAGCAGCTGCACAA
AGCCATGAAGCGCTACGCCCTGGTGCCCGGCACCATCGCCTTTACCGACGCACATATCGAGGTGGACATTACCTACGCCGAGTACT
TCGAGATGAGCGTTCGGCTGGCAGAAGCTATGAAGCGCTATGGGCTGAATACAAACCATCGGATCGTGGTGTGCAGCGAGAATAGC
TTGCAGTTCTTCATGCCCGTGTTGGGTGCCCTGTTCATCGGTGTGGCTGTGGCCCCAGCTAACGACATCTACAACGAGCGCGAGCT
GCTGAACAGCATGGGCATCAGCCAGCCCACCGTCGTATTCGTGAGCAAGAAAGGGCTGCAAAAGATCCTCAACGTGCAAAAGAAGC
TACCGATCATACAAAAGATCATCATCATGGATAGCAAGACCGACTACCAGGGCTTCCAAAGCATGTACACCTTCGTGACTTCCCAT
TTGCCACCCGGCTTCAACGAGTACGACTTCGTGCCCGAGAGCTTCGACCGGGACAAAACCATCGCCCTGATCATGAACAGTAGTGG
CAGTACCGGATTGCCCAAGGGCGTAGCCCTACCGCACCGCACCGCTTGTGTCCGATTCAGTCATGCCCGCGACCCCATCTTCGGCA
ACCAGATCATCCCCGACACCGCTATCCTCAGCGTGGTGCCATTTCACCACGGCTTCGGCATGTTCACCACGCTGGGCTACTTGATC
TGCGGCTTTCGGGTCGTGCTCATGTACCGCTTCGAGGAGGAGCTATTCTTGCGCAGCTTGCAAGACTATAAGATTCAATCTGCCCT
GCTGGTGCCCACACTATTTAGCTTCTTCGCTAAGAGCACTCTCATCGACAAGTACGACCTAAGCAACTTGCACGAGATCGCCAGCG
GCGGGGCGCCGCTCAGCAAGGAGGTAGGTGAGGCCGTGGCCAAACGCTTCCACCTACCAGGCATCCGCCAGGGCTACGGCCTGACA
GAAACAACCAGCGCCATTCTGATCACTCCAGAAGGGGTTTAAGACTGGGATCCGCCCCTCTCCCTCCCCCCCCCCTAACGTTACTG
GCCGAAGCCGCTTGGAATAAGGCCGGTGTGCGTTTGTCTATATGTTATTTTCCACCATATTGCCGTCTTTTGGCAATGTGAGGGCC
CGGAAACCTGGCCCTGTCTTCTTGACGAGCATTCCTAGGGGTCTTTCCCCTCTCGCCAAAGGAATGCAAGGTCTGTTGAATGTCGT
GAAGGAAGCAGTTCCTCTGGAAGCTTCTTGAAGACAAACAACGTCTGTAGCGACCCTTTGCAGGCAGCGGAACCCCCCACCTGGCG
ACAGGTGCCTCTGCGGCCAAAAGCCACGTGTATAAGATACACCTGCAAAGGCGGCACAACCCCAGTGCCACGTTGTGAGTTGGATA
GTTGTGGAAAGAGTCAAATGGCTCTCCTCAAGCGTATTCAACAAGGGGCTGAAGGATGCCCAGAAGGTACCCCATTGTATGGGATC
TGATCTGGGGCCTCGGTACACATGCTTTACATGTGTTTAGTCGAGGTTAAAAAAACGTCTAGGCCCCCCGAACCACGGGGACGTGG
TTTTCCTTTGAAAAACACGATGATAATATGGCCACAGCCACCATGCCAGAGCCAGCGAAGTCTGCTCCCGCCCCGAAAAAGGGCTC
CAAGAAGGCGGTGACTAAGGCGCAGAAGAAAGGCGGCAAGAAGCGCAAGCGCAGCCGCAAGGAGAGCTATTCCATCTATGTGTACA
AGGTTCTGAAGCAGGTCCACCCTGACACCGGCATTTCGTCCAAGGCCATGGGCATCATGAATTCGTTTGTGAACGACATTTTCGAG
CGCATCGCAGGTGAGGCTTCCCGCCTGGCGCATTACAACAAGCGCTCGACCATCACCTCCAGGGAGATCCAGACGGCCGTGCGCCT
GCTGCTGCCTGGGGAGTTGGCCAAGCACGCCGTGTCCGAGGGTACTAAGGCCATCACCAAGTACACCAGCGCTAAGGATCCCCGGG
TACCGGTCGCCACCATGGTGAGCAAGGGCGAGGAGCTGTTCACCGGGGTGGTGCCCATCCTGGTCGAGCTGGACGGCGACGTAAAC
GGCCACAAGTTCAGCGTGTCCGGCGAGGGCGAGGGCGATGCCACCTACGGCAAGCTGACCCTGAAGTTCATCTGCACCACCGGCAA
GCTGCCCGTGCCCTGGCCCACCCTCGTGACCACCCTGACCTGGGGCGTGCAGTGCTTCGCCCGCTACCCCGACCACATGAAGCAGC
ACGACTTCTTCAAGTCCGCCATGCCCGAAGGCTACGTCCAGGAGCGCACCATCTTCTTCAAGGACGACGGCAACTACAAGACCCGC
GCCGAGGTGAAGTTCGAGGGCGACACCCTGGTGAACCGCATCGAGCTGAAGGGCATCGACTTCAAGGAGGACGGCAACATCCTGGG
GCACAAGCTGGAGTACAACGCCATCAGCGACAACGTCTATATCACCGCCGACAAGCAGAAGAACGGCATCAAGGCCAACTTCAAGA
TCCGCCACAACATCGAGGACGGCAGCGTGCAGCTCGCCGACCACTACCAGCAGAACACCCCCATCGGCGACGGCCCCGTGCTGCTG
CCCGACAACCACTACCTGAGCACCCAGTCCAAGCTGAGCAAAGACCCCAACGAGAAGCGCGATCACATGGTCCTGCTGGAGTTCGT
GACCGCCGCCGGGATCACTCTCGGCATGGACGAGCTGTACAAGGAGGGCCGCGGCAGCCTGCTGACCTGCGGCGACGTGGAGGAAA
ACCCCGGCCCCATGACCGAGTACAAGCCCACGGTGCGCCTCGCCACCCGCGACGACGTCCCCCGGGCCGTACGCACCCTCGCCGCC
GCGTTCGCCGACTACCCCGCCACGCGCCACACCGTCGACCCGGACCGCCACATCGAGCGGGTCACCGAGCTGCAAGAACTCTTCCT
CACGCGCGTCGGGCTCGACATCGGCAAGGTGTGGGTCGCGGACGACGGCGCCGCGGTGGCGGTCTGGACCACGCCGGAGAGCGTCG

AAGCGGGGGCGGTGTTCGCCGAGATCGGCCCGCGCATGGCCGAGTTGAGCGGTTCCCGGCTGGCCGCGCAGCAACAGATGGAAGGC
CTCCTGGCGCCGCACCGGCCCAAGGAGCCCGCGTGGTTCCTGGCCACCGTCGGCGTGTCGCCCGACCACCAGGGCAAGGGTCTGGG
CAGCGCCGTCGTGCTCCCCGGAGTGGAGGCGGCCGAGCGCGCCGGGGTGCCCGCCTTCCTGGAGACCTCCGCGCCCCGCAACCTCC
CCTTCTACGAGCGGCTCGGCTTCACCGTCACCGCCGACGTCGAGGTGCCCGAAGGACCGCGCACCTGGTGCATGACCCGCAAGCCC
GGTGCCTGA
